# Pharmacodynamics of mutant-*IDH1* inhibitors in glioma patients probed by in vivo 3D MRS imaging of 2-hydroxyglutarate

**DOI:** 10.1038/s41467-018-03905-6

**Published:** 2018-04-16

**Authors:** Ovidiu C. Andronesi, Isabel C. Arrillaga-Romany, K. Ina Ly, Wolfgang Bogner, Eva M. Ratai, Kara Reitz, A. John Iafrate, Jorg Dietrich, Elizabeth R. Gerstner, Andrew S. Chi, Bruce R. Rosen, Patrick Y. Wen, Daniel P. Cahill, Tracy T. Batchelor

**Affiliations:** 1 Department of Radiology, Massachusetts General Hospital, Athinoula A. Martinos Center for Biomedical Imaging, Harvard Medical School, Boston, MA 02129 USA; 2 Department of Neurology, Massachusetts General Hospital, Stephen E. and Catherine Pappas Center for Neuro-Oncology, Division of Hematology/Oncology, Harvard Medical School, Boston, MA 02114 USA; 3 Department of Biomedical Imaging and Image-guided Therapy, High Field MR Centre, Medical University of Vienna, Vienna, 1090 Austria; 4 Department of Neurosurgery, Massachusetts General Hospital, Harvard Medical School, Boston, MA 02114 USA; 5 Department of Pathology, Massachusetts General Hospital, Center for Integrated Diagnostics, Harvard Medical School, Boston, MA 02114 USA; 6 Brain Tumor Center, Laura and Isaac Perlmutter Cancer Center, New York University Langone Medical Center and School of Medicine, New York, NY 10016 USA; 7 Dana-Farber Cancer Institute, Boston, MA 02284 USA

## Abstract

Inhibitors of the mutant isocitrate dehydrogenase 1 (*IDH1*) entered recently in clinical trials for glioma treatment. Mutant *IDH1* produces high levels of 2-hydroxyglurate (2HG), thought to initiate oncogenesis through epigenetic modifications of gene expression. In this study, we show the initial evidence of the pharmacodynamics of a new mutant *IDH1* inhibitor in glioma patients, using non-invasive 3D MR spectroscopic imaging of 2HG. Our results from a Phase 1 clinical trial indicate a rapid decrease of 2HG levels by 70% (CI 13%, *P* = 0.019) after 1 week of treatment. Importantly, inhibition of mutant *IDH1* may lead to the reprogramming of tumor metabolism, suggested by simultaneous changes in glutathione, glutamine, glutamate, and lactate. An inverse correlation between metabolic changes and diffusion MRI indicates an effect on the tumor-cell density. We demonstrate a feasible radiopharmacodynamics approach to support the rapid clinical translation of rationally designed drugs targeting *IDH1/2* mutations for personalized and precision medicine of glioma patients.

## Abstract (web summary)

Inhibitors of mutant isocitrate dehydrogenase 1 (IDH1) entered recently clinical trials for treatment of gliomas. Here, the authors apply a MRS imaging method for 2HG detection and assessement of the pharmacodynamic effects of the mutant IDH1 inhibitor (IDH305) in 8 mutant IDH1 glioma patients.

## Introduction

Recurrent heterozygous mutations in the isocitrate dehydrogenase 1 (*IDH1*) enzyme are very common in grade II-III glioma and secondary glioblastoma^1,2^, which now are recognized in the 2016 World Health Organization classification of central nervous system tumors^3^ to be a particular subtype of glioma characterized by the distinct molecular and clinical phenotypes. The metabolic hallmark of this glioma subtype is de novo production of high levels of the oncometabolite 2-hydroxyglutarate (2HG), exclusively in the form of the D-enantiomer (D-2HG), by the mutant *IDH1* enzyme through the NADPH-dependent reduction of α-ketoglutarate (αKG)^4^. *IDH1* mutation is an early genetic event in gliomagenesis, and 2HG is believed to further promote tumorigenesis through the inhibition of αKG-dependent dioxygenases^5^ and chromatin modifiers, resulting in DNA-/histone-hypermethylation and loss of the epigenetic control of gene expression^6–10^.

Gliomas are rarely curable tumors with a low survival rate (34%) at 5 years (SEER, CBTRUS 2012). Although the mutant *IDH1* glioma are more amenable to gross-total resection^11^ and seem to respond better to standard chemoradiation^12–14^ especially  when associated 1p/19q co-deletion, the *IDH1* mutations represent a clear opportunity for more targeted treatment either by small-molecule inhibitors of the mutant *IDH1* enzyme^15^, immunotherapy^16^, or by synthetic lethality strategies^17,18^. Recently, mutant *IDH1* targeted therapeutic strategies have entered Phase I clinical trials, and neuroimaging can accelerate the clinical translation of these treatments^19–21^. Preliminary data from the clinical trials in acute myeloid leukemia (AML) suggest that there is benefit of mutant *IDH2* inhibition and lowering of 2HG concentration^22^, however no data are yet available from clinical trials in mutant *IDH1* glioma patients. Our study sheds light on the metabolic effects in response to mutant *IDH1* inhibition in glioma patients.

The unique biology of 2HG makes this metabolite a very specific biomarker that can be used for the diagnostic, prognostic, prediction, and pharmacodynamics assessment by probing the tumor burden, cancer pathways, and treatment mechanisms in the mutant *IDH1* gliomas. 2HG can be detected noninvasively by in vivo magnetic resonance spectroscopy (MRS), and several methods^23–26^ have been demonstrated to resolve the spectral overlap between 2HG and other normally occurring brain metabolites. In particular, for monitoring the treatment in mutant *IDH1* glioma patients the non-invasive MRS detection of 2HG is more feasible^27,28^ and has clear advantages, compared to biopsies: (1) there are no associated risks, (2) the technique can be repeated multiple times, (3) the technique can probe multiple tumor regions, and (4) MRS can investigate normal appearing brain as internal control. Alternative methods^29–33^, such as measuring 2HG in blood, urine, and CSF samples have shown mixed results for mutant *IDH1* glioma, with some studies reporting elevated 2HG only in CSF^33^, while others found elevated 2HG only in urine^32^. The lack of uniform results may be related to lack of a standard protocol with differences in analytical methods, sample collection, and preservation that add to the biological variability. In addition, 2HG levels in periphery are diluted, the spatial localization is lost, tumor heterogeneity cannot be probed, and collecting CSF is not without complication, especially for longitudinal monitoring. Besides tumor production, 2HG levels in serum and urine are also influenced by other factors, such as the blood–brain barrier (BBB), which is less compromised in mutant *IDH1* glioma, and the shedding of tumor material may thus be reduced. The combination of all these factors make the detection of 2HG in CSF, serum, and urine less straightforward in mutant IDH glioma patients, compared to AML patients. On the other hand, MRS methods are rapid, easy to perform, and inexpensive, relative to genomics or other in vivo molecular imaging, such as PET or SPECT. 2HG imaging provides better specificity for detection of *IDH1* mutations than alternative MRI methods^34–39^, and could help distinguish true/pseudo-response in treatment assessment^40^. In addition, in the case of mutant *IDH1* inhibitors, 2HG as a direct pharmacodynamic biomarker is expected to probe earliest the target modulation, compared to either conventional anatomical magnetic resonance imaging, such as contrast enhanced T1-weigted and fluid attenuated inversion recovery (FLAIR) MRI that are part of RANO criteria^41,42^ or the more advanced diffusion/perfusion MRI^43^.

In this study we used a recently demonstrated 3D MRS imaging (MRSI) method for 2HG detection^27^ to assess the pharmacodynamic effects of the new investigational drug IDH305 (Novartis Pharmaceuticals) in mutant *IDH1* glioma patients enrolled in an open label first-in-human Phase I clinical trial (ClinicalTrials.gov identifier: NCT02381886). IDH305 is an orally available, brain penetrant, mutant-selective allosteric high affinity *IDH1* inhibitor that acts on both canonical (R132H) and non-canonical (R132C) mutated enzymes, but has much lower affinity for wild-type *IDH1* or mutant *IDH2* enzymes. IDH305 potently reduces the 2HG production in preclinical models, in which a single dose of 100 mg/kg is sufficient to reduce the tumor 2HG levels by 95% in nude mice with *IDH1* mutant flank tumors, and has shown in vitro antiproliferative effects (Novartis Pharmaceuticals Phase I clinical trial protocol, ClinicalTrials.gov identifier: NCT02381886). Hence, the biological effects of IDH305 in patients are expected to be restricted to tumor cells harboring *IDH1*-R132 mutations. Here, we show initial evidence regarding the pharmacodynamic effects of mutant *IDH1* inhibition in glioma patients. Our primary goal in this study was to probe whether IDH305 can modulate target activity in glioma patients. In addition to 2HG, we measured other metabolites that are coupled with 2HG production, such as glutamate, glutamine, glutathione, and lactate. We investigated the relation between the change in 2HG levels and other metabolites, and the correlation with other imaging parameters such as tumor volumetrics, and apparent diffusion coefficient (ADC). The scope of this analysis was to uncover the mechanisms that explain the action of the mutant *IDH1* inhibitors in glioma patients, and determine a comprehensive panel of imaging biomarkers that can be used to objectively assess the response to mutant *IDH1* inhibition. By combining genetics, pharmacology, and radiology, we demonstrate a comprehensive strategy, which we name *radiopharmacodynamics*, that can be used for precision and personalized medicine.

## Results

### Histological grading and molecular subtyping of patients

Eight patients were enrolled in Phase I clinical trial of IDH305 at our institution. Distribution of patients based on the 2016 WHO classification^3^ was two grade II, five grade III, and one grade IV. Table 1 lists the demographics, histological grading/typing and the molecular subtyping, FLAIR and 2HG levels at baseline for all the patients.

**Table 1:** Demographic, histology and molecular markers of patients

Pt	A/G	Grade	Type	*IDH1*	IDH305	1p/19q	TP53	MGMT	FLAIR (cm^3^)	2HG (mM)
#1	49/F	III	AOD	R132H	550 mg	co-del	n.a.	n.a.	15.9	2.32
#2	48/F	II	A	R132H	550 mg	n.d.	mut	n.a.	23.7	0.28
#3	30/F	IV	GBM	R132H	550 mg	co-del	n.a.	met	7.9	1.73
#4	42/M	III	AA	R132H	550 mg	not-del	mut	umet	10.3	0.59
#5	38/M	III	AA	R132H	550 mg	not-del	mut	umet	8.8	1.04
#6	49/F	III	AA	R132H	550 mg	co-del	n.a.	n.a.	43.2	1.31
#7	31/M	III	AA	R132H	900 mg	not-del	mut	n.a.	3.9	n.d.
#8	46/F	II	A*	R132H	550 mg	n.d.	n.a.	n.a.	7.8	n.d.

Abbreviations: *Pt *Patient number, *A/G* Age/Gender, *A* Astrocytoma, *AA *Anaplastic Astrocytoma, *AOD* Anaplastic Oligodendroglioma, *GBM* Glioblastoma, IDH1 Isocitrate Dehydrogenase 1, *R132H *Arginine 132 to Histidine, *IDH305* inhibitor of IDH1-R132H, *1p/19q *co-deletion of 1p and 19q chromosome arms, *TP53* Tumor Protein p53, *MGMTO*^6^-Methylguanine DNA Methyltransferase, *FLAIR *Fluid Attenuated Inversion Recovery, *2HG *2-hydroxyglutarate, *co-del* co-delited, *mut* mutated, *met* methylated, *umet* un-methylated, *n.a.*not available*, n.d.* not detectable. Patients 1–5 had baseline and follow-up MRSI scans, patient 6 had only baseline scan, and patients 7–8 had no detectable 2HG at baseline. *According to WHO 2007 this patient was diagnosed as ganglioglioma with atypical features.

### Longitudinal metabolic imaging of patients

Longitudinal imaging data was available from five patients (#1–5), all receiving 500 mg of IDH305 orally BID. One patient (#6) had only baseline imaging and did not complete the post treatment scan, while in two other patients (#7 with grade II diffuse astrocytoma, and #8 with grade III anaplastic astrocytoma) the 2HG levels at pretreatment imaging scan were not detectable by MRSI. These two patients (#7 and #8) had the smallest FLAIR volumes among all patients, 3.8 and 7.8 cm^3^, respectively. The 2HG imaging results from these three patients (#6–8) are shown in Supplementary Figure 1. A recent study has found that MRS detection of 2HG is significantly reduced in patients with FLAIR volumes smaller than 8 cm^3^. The patient with 7.8 cm^3^ FLAIR volume had the shortest time interval (2 weeks) between the surgery and the baseline scan, with visible blood products in the surgical cavity. The tumor location in both patients was in a brain region which yielded good quality MRSI (SNR > 5, FWHM < 12 Hz).

**Fig. 1:**
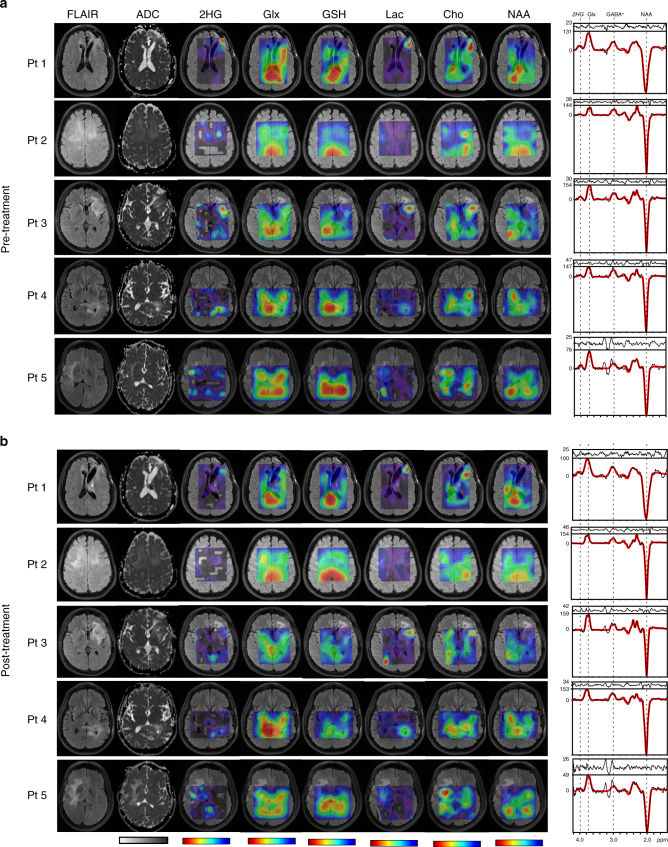
Longitudinal imaging in mutant *IDH1* glioma patients treated with IDH305 inhibitor. **a** Baseline anatomical, structural and metabolic maps before treatment. **b** Follow-up anatomical, structural, and metabolic maps after 1 week of IDH305 treatment. Metabolic maps are converted to mM by reference to healthy creatine levels. The color bars ranges are 0–3 mM (2HG), 0–20 mM (Glx), 0–1 mM (GSH), 0–16 mM (Lac), 0–3 mM (tCho), 0–20 mM (NAA), and 0–4 μm^2^/ms (ADC). On the right, examples of 2HG edited spectra (black trace) and the LCModel fits (red trace) are shown

Figure 1 shows longitudinal 3D imaging of 2HG and other brain metabolites in glioma patients (#1–5) before and after 1 week of mutant *IDH1* inhibitor treatment with the experimental drug IDH305. Anatomical FLAIR and structural diffusion MR imaging acquired at the same time points are also presented. In the five patients with longitudinal 2HG imaging, the highest levels of 2HG are obtained in the region of FLAIR abnormality at the baseline scan. In some cases, high 2HG levels are noticed further away from the FLAIR region. However, by imposing a threshold on the goodness of fit (Cramer Rao Lower Bounds, CRLB < 25%) for 2HG signal, we obtain the mask with reliable 2HG quantification, as shown in Supplementary Figure 2 where the MRSI voxels remote from the tumor are eliminated.

### Tumor 2HG levels decrease during mutant IDH1 inhibition

**Fig. 2:**
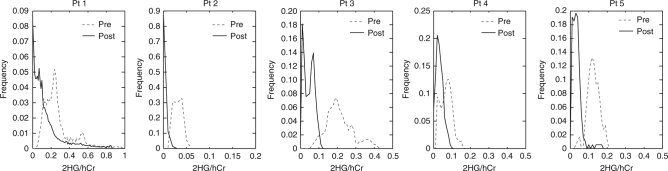
Histograms of 2HG levels. The distribution of patient 2HG levels within the tumor ROI are shown before (dashed line) and after 1 week (continuous line) of treatment with the mutant *IDH1* inhibitor IDH305

The distribution of 2HG values over the tumor volume and how this changed with the treatment was compared using histogram analysis. Histograms of tumor 2HG levels before and after treatment are shown in Fig. 2. The pretreatment histogram distributions are wider with a right tail toward higher values, while the left tail does not include values that are close to zero. The posttreatment histograms are narrower, shifted toward the left, and are asymmetric having the largest contribution toward zero.

**Fig. 3:**
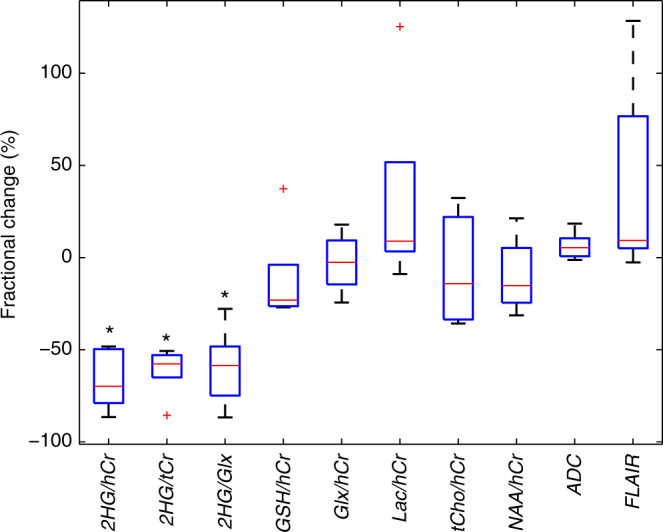
Boxplot of the fractional changes for the mean values averaged over the tumor ROI. Box size represents the first and third quartiles, and horizontal red line indicates the median. Statistical significant changes are indicated by the * symbol (*P* < 0.05)

The fractional change of mean values averaged over the entire tumor region of interest (ROI) is shown in Fig. 3 for 2HG, other brain metabolites, metabolic ratios, the apparent diffusion coefficient (ADC), and the ROI volume. After 1 week of treatment only the levels of 2HG show statistically significant (*P* < 0.05) changes with 70% reduction of the 2HG/hCr ratio (2HG relative to healthy creatine), 57% reduction of the 2HG/tCr ratio (2HG relative to tumor creatine), and 58% reduction of the 2HG/Glx ratio (2HG relative to glutamine + glutamate). There was a trend for an increase in lactate levels (9%), increase in ADC (5%), and increase in FLAIR volume (9%). The median and confidence intervals of pre and posttreatment mean values across five patients are listed in Table 2.

**Table 2:** Quantification of metabolites and imaging parameters

	**2HG/** **hCr**	**2HG/** **tCr**	**2HG/** **Glx**	**GSH/** **hCr**	**Glx/** **hCr**	**Lac/** **hCr**	**tCho/** **hCr**	**NAA/** **hCr**	**ADC (mm** ^**2**^ **/ms)**	**FLAIR (cm** ^**3**^ **)**
**Pre (median)**	0.131	0.185	0.128	0.045	1.001	0.627	0.244	0.617	1.206	10.295
**Pre (95% CI)**	0.091	0.163	0.074	0.008	0.133	0.433	0.065	0.130	0.095	5.787
**Post (median)**	0.057	0.102	0.064	0.039	0.974	0.760	0.226	0.626	1.312	16.861
**Post (95% CI)**	0.043	0.078	0.057	0.006	0.217	0.314	0.087	0.220	0.096	3.951
***P*** **-value**	0.019	0.017	0.035	0.263	0.807	0.308	0.706	0.545	0.132	0.173

Median values, 95% Confidence Intervals (CI), and the *P*-value for the pre-treatment and post-treatment imaging biomarkers. Inhibitors of the mutant isocitrate dehydrogenase 1 (*IDH1*) entered recently in clinical trials for the treatment of gliomas. Here, the authors apply a MRS imaging method for 2HG detection and assessement of the pharmacodynamic effects of the mutant* IDH1* inhibitor (IDH305) in eight mutant* IDH1* glioma patients.

In three patients, we had a third follow-up MR scan performed at 1 month after the start of IDH305 therapy. The 2HG levels (2HG/hCr) at 1 month (Median = 0.06, CI = 0.07) and at 1 week (Median = 0.05, CI = 0.03) are similarly decreased, compared to the baseline (Median = 0.21, CI = 0.1). There is increased variability at 1 month because the 2HG levels drop toward the detectability threshold for MRS, hence the precision of our estimates decreases. We have included in Supplementary Figure 3 the 2HG imaging data for the third visit and in Supplementary Figure 4 the plot of 2HG values for all three time points.

### Metabolic reprogramming of mutant IDH1 glioma

Next, we investigated whether there is a relationship between the change in 2HG levels and the metabolites that are coupled with 2HG production. In particular, glutathione, glutamine, and glutamate are a part of the metabolic reactions that are closely connected with the mutant *IDH1* pathway. These metabolites have important cellular roles, GSH is the major scavenger of free radical species required to maintain the redox balance, and glutamine is an anaplerotic source of carbon and nitrogen in the brain. In addition to these, lactate is a metabolite of particular significance in cancer, related to the Warburg effect, and is implicated in many cancer mechanisms.

The increase in lactate levels shown in Fig. 3 may be an important observation associated with the inhibition of mutant *IDH1*. It has been reported that the activity of lactate dehydrogenase A (LDH-A) is downregulated in mutant *IDH1* glioma cells^44^, which is opposite to the typical upregulation of LDH-A in most other cancers.

**Fig. 4:**
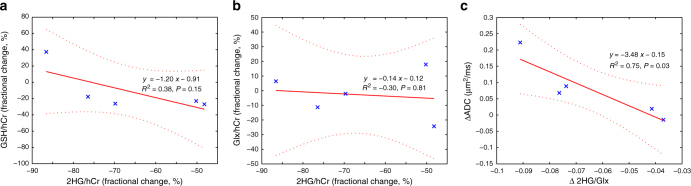
Relation between the treatment induced changes of 2HG and other metabolic and imaging biomarkers. Linear model fitting has been employed to investigate the relationship between the changes: **a** 2HG and GSH; **b** 2HG and Glx; **c** ratio of 2HG/Glx and ADC. A significant inverse correlation was found between changes of 2HG/Glx ratio and changes of ADC after 1 week of treatment with IDH305

The mutant *IDH1* couples the reduction of αKG to 2HG with the oxidation of NADPH to NADP^+^. It has been shown that wild-type *IDH1* is the most important source of NADPH in the brain, and the mutant *IDH1* enzyme consumes more NADPH than produced by the wild-type *IDH1* enzyme^45^. In turn, NADPH is used to regenerate the reduced glutathione, and a decrease in NADPH in mutant *IDH1* glioma may lower the GSH levels. Conversely, an increase in NADPH due to inhibition of mutant *IDH1* may result in an increase in GSH levels. A linear model fit in Fig. 4a shows a trend for inverse correlation between fractional changes of 2HG and GSH levels.

Glutamine is an important source for tricarboxylic acid cycle anaplerosis in cancer cells^46^, and in the case of mutant *IDH1* glioma cells, glutamine becomes the major source for 2HG synthesis via a two-step process, involving glutamate and αKG as intermediaries^4^. Glutamate and glutamine levels have been found to be lower in mutant *IDH1* compared to wild-type *IDH1* glioma patients, as measured by the mass spectrometry in tumor biopsies^47^. Previously, we found that Glx signal (glutamate + glutamine) by in vivo MRSI is decreased in tumor, compared to contralateral normal appearing white matter in mutant *IDH1* patients. Inhibition of mutant *IDH1* activity might limit the conversion of glutamine into 2HG. Our results from Fig. 4b indicate a trend for inverse correlation between the fractional changes of 2HG and Glx, however this correlation is weaker than in the case of GSH.

### Correlation of metabolic profile and cellular density

Previous studies^48^ have found that 2HG levels correlate with the cellular density of mutant *IDH1* glioma tumors. In this study, we sought to investigate whether the dynamic changes in 2HG levels would correlate with the dynamic changes in cell density during treatment with mutant *IDH1* inhibitors. Since longitudinal biopsies were not available, we correlated the changes in 2HG with the changes in an imaging parameter that is a surrogate biomarker for cell density. The apparent diffusion coefficient is an imaging biomarker that probes tissue microstructure and cell density^49,50^. ADC has been shown to increase in tumors that respond to the treatment^51,52^. The results of a linear model fit in Fig. 4c shows that there is a significant inverse correlation between the changes in the 2HG/Glx ratio and the changes in ADC. In the case of mutant *IDH1* inhibition, the simultaneous decrease of the 2HG/Glx ratio and the increase in ADC might be interpreted as a sign of objective treatment response in glioma patients.

## Discussion

Mutations of *IDH1* offer the opportunity to develop an effective treatment against gliomas. The efforts to translate mutant *IDH1*-targeted treatments are significantly enhanced by the existence of the 2HG biomarker that can be measured to assess the objective response rates. However, serial biopsies for pharmacodynamic assessments in glioma patients pose the risks of neurological injury or infection, which in the setting of clinical trials might not outweigh the clinical benefits. Hence, imaging^53^ and “liquid biopsies”^54^ (blood, urine, and cerebrospinal fluid) remain the only options, at present, to objectively monitor the treatments over time in glioma trials. 2HG is the most informative biomarker on the pharmacodynamics of mutant *IDH1* inhibitors. The intra-tumoral 2HG levels probe directly the in vivo activity of mutant *IDH1* enzyme, while the peripheral 2HG levels are modulated in addition by other factors, such as the BBB. In particular, 3D imaging is well-suited to serially monitor the treatment because it can investigate the entire tumor volume and healthy brain, and it is not affected by sampling bias of single-point measurements. Hence, precision medicine-based clinical trials become feasible in glioma patients, by employing quantitative imaging for pharmacodynamic biomarker assessment in longitudinal studies.

The molecular mechanisms of the mutant *IDH1* glioma are not fully understood, and the implications of mutant *IDH1* inhibition are still a matter of debate. Earlier, preclinical data demonstrated^15^ that the mutant *IDH1* inhibition might be able to revert the histone hypermethylation, but not DNA hypermethylation, induce cell differentiation, and delay tumor growth. Recently, preclinical studies using multiple patient-derived glioma models have failed to show an effect on tumor growth by mutant *IDH1* inhibitors^18^. More importantly, the clinical data show that mutant *IDH1* patients have significantly longer survival compared to wild-type *IDH1* patients^2^, implying some benefit in harboring the *IDH1* mutations either by a more indolent progression or intrinsic vulnerabilities that cause better response to existing therapies^14^.

To unequivocally answer the conundrum regarding the implications of mutant *IDH1* inhibition, it is paramount to probe the target modulation in patients and exclude the lack of in vivo drug activity. Our results demonstrate that IDH305 modulates target activity and is effective in the inhibition of mutant *IDH1*, lowering 2HG levels by 70% after 1 week of treatment. In addition, there are other metabolic effects of mutant *IDH1* inhibition, as suggested by a trend toward an inverse correlation between changes of 2HG and glutathione or glutamine + glutamate, respectively. Opposite changes of 2HG and Glx levels in mutant *IDH1* glioma (i.e., high 2HG and low Glx tumor levels) may make the 2HG/Glx ratio a metabolic biomarker that has an increased range to detect mutant *IDH1* tumor tissue. Although similar to the Cho/NAA ratio (i.e., high Cho and low NAA tumor levels) in wild-type gliomas, the 2HG/Glx ratio may be more specific to detect the targeted treatment response against mutant *IDH1* gliomas. Changes in glutathione levels are particularly relevant in connection with tumor susceptibility to radiotherapy. The biological effects of radiotherapy are amplified by free radical oxygen species, which in turn are neutralized by glutathione. Hence, it has been hypothesized that mutant *IDH1* patients having lower NADPH, and consequently lower GSH levels, would be more radiosensitive^45,55^. On the other hand, an increase in GSH due to m*IDH1* inhibition would diminish the radiobiological effects, and may argue against the simultaneous administration of mutant *IDH1* inhibitors and radiotherapy^56^. The correlation between changes of Glx and 2HG is weaker than the one between the changes of GSH and 2HG, which may be explained by the fact that glutamine is used in many metabolic pathways, including being a precursor for the synthesis of GSH. The increase in lactate levels in post-treatment scans could indicate that downregulation of the LDH-A activity is reversible through mutant *IDH1* inhibition. Lactate is thought to play many roles in the pathophysiology of cancer, creating an acidic environment that favors cell migration, immune escape, and epigenetic alterations^57^. On the other hand, we observed a significant correlation between decreasing levels of 2HG/Glx and increasing ADC values. An increase in ADC is associated with a decrease in cellular density, and is regarded as a positive outcome in cancer treatment. Thus, raising lactate levels and increasing ADC values by mutant *IDH1* inhibition poses a puzzling scenario.

In some patients, we noted an increase of the FLAIR volume which raises the question whether these patients experienced tumor progression during the treatment with IDH305. A related question is whether the quantification of 2HG levels is affected by the change in FLAIR volumes. In order to avoid the possible confounding effect due edema and changes in water content in tumor, we quantified 2HG and the other tumor metabolites relative to the levels of creatine in the normal appearing (healthy) brain which is not affected by IDH305, and where no change in FLAIR is noticed. Tumor 2HG levels also decrease when quantified, relative to healthy creatine, which indicate a true 2HG decrease. A true 2HG decrease and the decrease of total choline would reject the tumor progression and would be consistent with pseudo-progression radiographic changes. In addition, no worsening of clinical symptoms, such as progressive headaches were reported by the patients during the course of the study.

Metabolic maps of the mutant IDH glioma patients show that levels of glutamate/glutamine (Glx) and glutathione (GSH) are decreased in the tumor regions compared to the contralateral regions, while lactate is increased in the tumor. However, while 2HG and lactate are focally increased in the tumor, because of lack of background signal in the surrounding tissue, the maps of Glx and GSH show smaller contrast between the tumor and the healthy tissue due to larger background signal of the normal brain. In addition, the longitudinal changes of Glx could be confounded by different fate of glutamate and glutamine in tumor metabolism. Methods^58–61^ that can separate better the contributions of Glu and Gln in MR spectroscopy might be able to differentiate better whether the inhibition of 2HG in patients is coupled with biochemical pathways of Gln or Glu.

Several limitations exist in our study, the most important being the low number of patients. Phase I clinical trials focused on the safety/toxicity/feasibility are performed on a small cohort of patients, and our imaging data is inherently limited by the design of the Phase I study. In addition, recurrent glioma patients enrolled in the Phase I trial are particularly challenging for 2HG detection, since these patients have a history of multiple prior therapies, including surgical resections. Existing data^27,28^ indicate that current chemoradiation therapies are also effective in reducing 2HG levels. Hence, it is expected that the baseline 2HG levels in patients from the Phase I study would be lower than in newly diagnosed patients. In our study, we were not able to detect 2HG levels at baseline in 25% (2/8) of the patients, which was related to small residual tumor postsurgery. With our MRSI protocol, we can reliably detect 2HG concentrations above 1 mM^62^, though lower concentrations (0.1–1 mM) are possible for data of very good spectral quality (linewidth < 6 Hz). Assuming 2HG concentration of 1 mM, tumors that have a volume of 1.9 cm^3^ or larger can be detected with our current protocol, but smaller tumors may be detected if they produce higher 2HG levels, or by more data averaging and longer acquisition times. These limitations due to imaging protocol mandates, further the efforts to improve the sensitivity of MRS-based 2HG detection. Another limitation is related to the fact that we do not yet have survival data to correlate with imaging parameters. Hence, we are not able, at this moment, to make any analysis regarding the predictive value of the objective response assessment based on 2HG levels, nor to infer any conclusion regarding the patient benefit. Despite these limitations, our preliminary results from this Phase I study shed light on the metabolic networks that may be impacted in patients by the inhibition of mutant *IDH1*, and potentially guide the future development. Although interesting, our imaging results need to be replicated in a larger cohort of patients.

Our *radiopharmacodynamics* study demonstrates the feasibility of image-based 2HG pharmacodynamic serial assessments in patients treated with mutant *IDH1* inhibitors. Since no animal model entirely recapitulates the human disease, there is no better substitute for human studies to test these drugs. A precision medicine approach is easy to perform by non-invasive longitudinal imaging in glioma patients where serial biopsies have high risks. Although, we found correlation between 2HG levels and widely used imaging parameters, such as ADC, the 2HG measurements are indispensable to probe the target modulation, mechanisms of action, metabolic reprogramming, and further could be used to study the dose-response activity of mutant *IDH1* inhibitors.

2HG measurements have the highest specificity for mutant *IDH1* mutations, and may distinguish true/pseudo-response/progression in the case of standard chemoradiation. However, we would like to caution that in the case of targeted mutant *IDH1* inhibition the interpretation and role of 2HG imaging needs to be carefully evaluated and understood. For example, mutant *IDH1* inhibition could efficiently reduce the 2HG levels, but this may not equate with tumor cell death. This is a situation analogous to that of anti-angiogenic therapy that reduces the contrast enhancement making tumors almost invisible on T1-weighted MRI, although gliomas can persist and progress behind a restored BBB^40^. Thus, decreased 2HG in the setting of mutant *IDH1* inhibition could be similar to reduced BBB leakiness of Gd-based contrast agents in the setting of anti-angiogenic therapy, and the pseudo-response by 2HG imaging might not be excluded. Nevertheless, the possibility of a 2HG imaging pseudo-response is likely to happen only in the case of mutant *IDH1* inhibitors used as monotherapy. In the case of standard chemoradiation, immunotherapy, or combination therapies, a decrease in 2HG is likely to reflect a decrease in tumor cell numbers. On the other hand, an increase of 2HG can rule out pseudo-progression and rule in true-progression. It is interesting to point that our data shows a tendency for larger FLAIR volumes after mutant *IDH1* inhibition, which may suggest either pseudo-progression or true-disease progression. The simultaneous increase of ADC with decrease of 2HG supports, in this case, the pseudo-progression interpretation of imaging findings. 2HG is likely the earliest biomarker of response in the case of mutant *IDH1* inhibition, and a disconnect between 2HG and conventional anatomical imaging is expected. To account for such a complex scenario, a larger panel of imaging biomarkers should be used in addition to 2HG, including other metabolites such as lactate, Glx, choline, NAA, and structural parameters such as ADC.

In summary, we demonstrate that 2HG imaging can inform the phase I clinical trial on the biological effects of targeted therapies against mutant *IDH1* gliomas, and potentially it could be used to guide future trial designs. An early assessment of treatment is important to avoid the possible negative side effects, and steer the course for maximizing patient benefit and quality of life. We expect that our methodology will help efforts of cancer scientists for further rational drug development.

## Materials and methods

### Patient population

Patients were enrolled in the imaging study, according to the inclusion/exclusion criteria of the Phase I clinical trial (ClinicalTrials.gov identifier: NCT02381886). Briefly, to be included the patients had to have confirmed *IDH1*-R132 mutant glioma for whom no curative therapy is available and who have evidence of disease progression following a standard therapy. Patients were excluded if they had less than 2 weeks from the last dose of systemic antineoplastic therapy, 6 weeks from bevacizumab, 3 months from radiotherapy, and less than 2 weeks from major surgery. In seven patients the IDH305 was administered at a dose of 550 mg orally twice a day (BID), while in one patient the dose was 900 mg orally BID. Imaging was performed at baseline 1–7 days prior to the start of IDH305, and at follow-up after 7 days of IDH305. The choice of the follow-up time point at 1 week was determined based on the following reasons: (1) a rapid inhibition of m*IDH1* by IDH305 with a large effect size on 2HG levels and (2) unknown toxicity that may limit the duration of IDH305 administration.

### IDH1 mutations

*IDH1*-mutational status was first tested by immunohistochemistry (IHC) analysis using an anti-human R132H antibody (DIANOVA)^63^ and subsequently confirmed by genetic sequencing (SNaPshot)^64,65^. Other molecular markers such as 1p/19q codeletion^66^ and MGMT promoter methylation^67^ were tested using fluorescence in situ hybridization (FISH) and polymerase chain reaction (PCR), respectively^64^, while TP53 mutation was confirmed also by SNaPshot^64,65^.

### Imaging protocol

MRI and MRSI data were acquired on a clinical 3T MR scanner (Tim Trio, Siemens, Erlangen) with a 32-channel head coil. A recently developed spectral-editing 3D MRSI sequence was used for metabolic imaging as following. Two spectral editing 3D MRSI acquisitions were performed (1) for 2HG detection^27^ with MEGA editing pulses applied at 1.9 ppm (ON) and 7.5 ppm (OFF) and (2) for GSH detection^68^ with MEGA editing pulses applied at 4.57 ppm (ON) and −1 ppm. Other acquisition parameters of 3D MRSI were: MEGA-LASER spectral editing, spiral k-space readout, TR = 1600 ms, TE = 68 ms, FOV = 200 × 200 × 200 mm^3^, VOI = 100 × 80 × 50 mm^3^, acquisition matrix 10 × 10 × 10 which was zero filled to 16 × 16 × 16 in the FFT reconstruction, yielding a digital voxel volume of 1.9 cm^3^, acquisition time 9:55 min:s. Second order B0 shimming was performed based on B0 fieldmaps obtained with a double echo gradient sequence. Water suppression was performed with WET scheme^69^. Anatomical and structural MRI was performed using FLAIR (TR/TI/TE = 10000/2500/70 ms, FOV = 220 × 186 × 138 mm^3^, matrix = 256 × 192 × 23) and diffusion tensor imaging (TR/TE = 7980/84 ms, *b*-values = 0/700 s/mm^2^, gradient directions = 12, FOV = 236 × 236 × 118 mm^3^, matrix = 128 × 128 × 64). To reproduce the positioning across visits for each patient, the online automatic slice positioning (AAscout^70^) was used during acquisition of all imaging data.

### Image analysis

Difference and OFF spectra from the 3D MRSI acquisition were fitted with LCModel^71^ software using the corresponding simulated basis sets for the MEGA-LASER sequence. Difference spectra were used to quantify 2HG, Glx (glutamate and glutamine), and glutathione (GSH). The OFF spectra were used to quantify the total choline (tCho), creatine (Cr), lactate (Lac), and N-acetyl-aspartate (NAA). Linewidth less than 0.1 ppm (12 Hz) and Cramer-Rao lower bound (CRLB) less than 25% was considered for goodness of fit of 2HG, Glx, and GSH in difference spectra. CRLB less than 20% was considered for the goodness of fit of tCho, Cr, Lac, and NAA in OFF spectra. The 3D metabolic maps were reconstructed using a combination of MINC (Montreal Neurological Institute), FSL (FMRIB Software Library, Oxford, UK), and MATLAB (Mathworks, Natick, MA) software tools. For all metabolites, relative maps were calculated by dividing the metabolite to healthy creatine (hCr) averaged in a ROI situated in contralateral normal appearing white matter. Healthy creatine levels were assumed to be 8 mM, and this was used to convert the units of the relative metabolic maps to mM concentration units. For 2HG, relative maps were calculated also relative to the creatine (2HG/tCr) and Glx in the tumor. Tumor ROIs were manually outlined around the hyperintense areas in the FLAIR images. Healthy ROIs were drawn in contralateral hemisphere in a region that appeared normal on FLAIR images. Using the upper threshold for CRLB (<25%), we obtained the ROI masks that select the voxels with reliable 2HG quantification. The ROIs for tumor, normal appearing white matter, and the 2HG goodness of fit mask (CRLB < 25%) are shown in Supplementary Figure 1.

### Statistical analysis

Differences in imaging biomarkers between post- and pre-treatment scans were deemed significant at a level of *P* < 0.05 using two-sided paired Student’s *t*-test. The data normality was verified with Kolmogorov-Smirnov test. Correlations between 2HG and other imaging biomarkers were calculated using Pearson product-moment correlation coefficient. Linear model fit was employed to establish the relation between 2HG and other biomarkers. All statistical analysis was performed with the statistical package of MATLAB.

### Data and materials availability

The datasets generated during and/or analyzed during the current study are available from the corresponding author on reasonable request.

## Electronic supplementary material

Supplementary Information
