# Supplementary material for: Pharmacodynamics of mutant-IDH1 inhibitors in glioma patients probed by in vivo 3D MRS imaging of 2-hydroxyglutarate
Source: Nat Commun. 2018 Apr 16;9:1474. doi: 10.1038/s41467-018-03905-6 (PMC5902553; doi:10.1038/s41467-018-03905-6)
Supplement: Supplementary file 1 — Supplementary Information [file 41467_2018_3905_MOESM1_ESM.pdf]

Pharmacodynamics of mutant-IDH1 inhibitors in glioma patients probed by *in-vivo* 3D MRS imaging of 2-hydroxyglutarate

Ovidiu C. Andronesi[1]*, Isabel C. Arrillaga-Romany[2], K. Ina Ly[2], Wolfgang Bogner[3], Eva M. Ratai[1], Kara Reitz[6], A. John Iafrate[4], Jorg Dietrich[2], Elizabeth R. Gerstner[2], Andrew S. Chi[5], Bruce R. Rosen[1], Patrick Y. Wen[7], Daniel P. Cahill[6], Tracy T. Batchelor[2]

(1) Athinoula A. Martinos Center for Biomedical Imaging, Department of Radiology, Massachusetts General Hospital, Harvard Medical School, Boston, MA, USA;
(2) Stephen E. and Catherine Pappas Center for Neuro-Oncology, Division of Hematology/Oncology, Department of Neurology, Massachusetts General Hospital, Harvard Medical School, Boston, MA, USA.
(3) High Field MR Centre, Department of Biomedical Imaging and Image-guided Therapy, Medical University of Vienna, Vienna, Austria
(4) Center for Integrated Diagnostics, Department of Pathology, Massachusetts General Hospital, Harvard Medical School, Boston, MA, USA;
(5) Brain Tumor Center, Laura and Isaac Perlmutter Cancer Center, New York University Langone Medical Center and School of Medicine, New York, NY 10016
(6) Department of Neurosurgery, Massachusetts General Hospital, Harvard Medical School, Boston, MA, USA;

(7) Dana-Farber Cancer Institute, Boston, MA, USA;

***Corresponding Author**:
Ovidiu C. Andronesi, MD, PhD, Martinos Center for Biomedical Imaging, Department of Radiology, Massachusetts General Hospital, Thirteenth Street, Charlestown, MA 02129, USA;
Email:oandronesi@mgh.harvard.edu

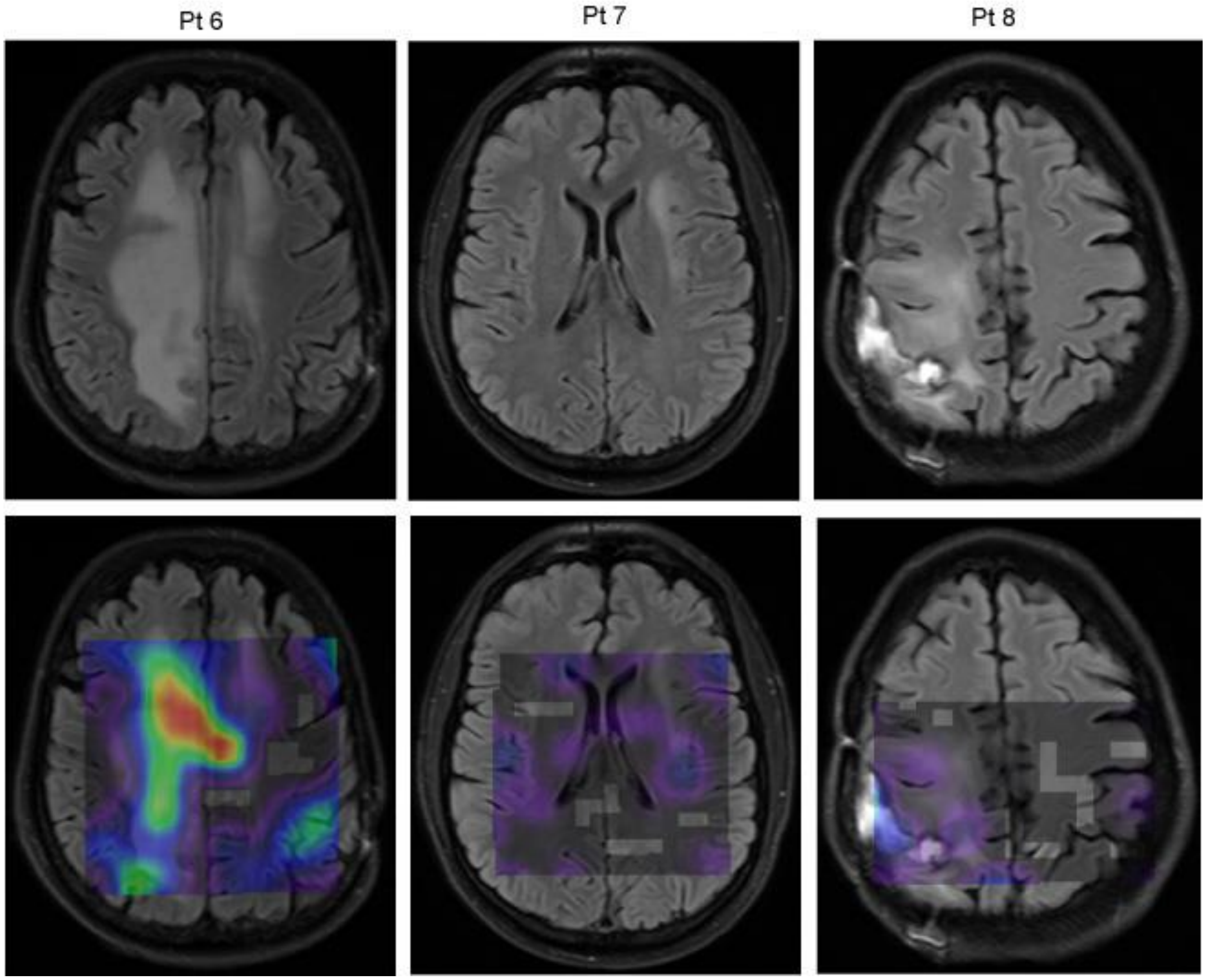

Supplementary Figure 1. 2HG maps and FLAIR images for three patients not included in the longitudinal analysis. Patient 6 had detectable 2HG levels at baseline but did not have any follow-up scan. Patients 7 and 8 had no clearly detectable tumor 2HG levels at baseline, which were comparable with the background levels in the normal appearing white matter. In patient 8 the brightest signal in the FLAIR image is due to blood products post-surgery.

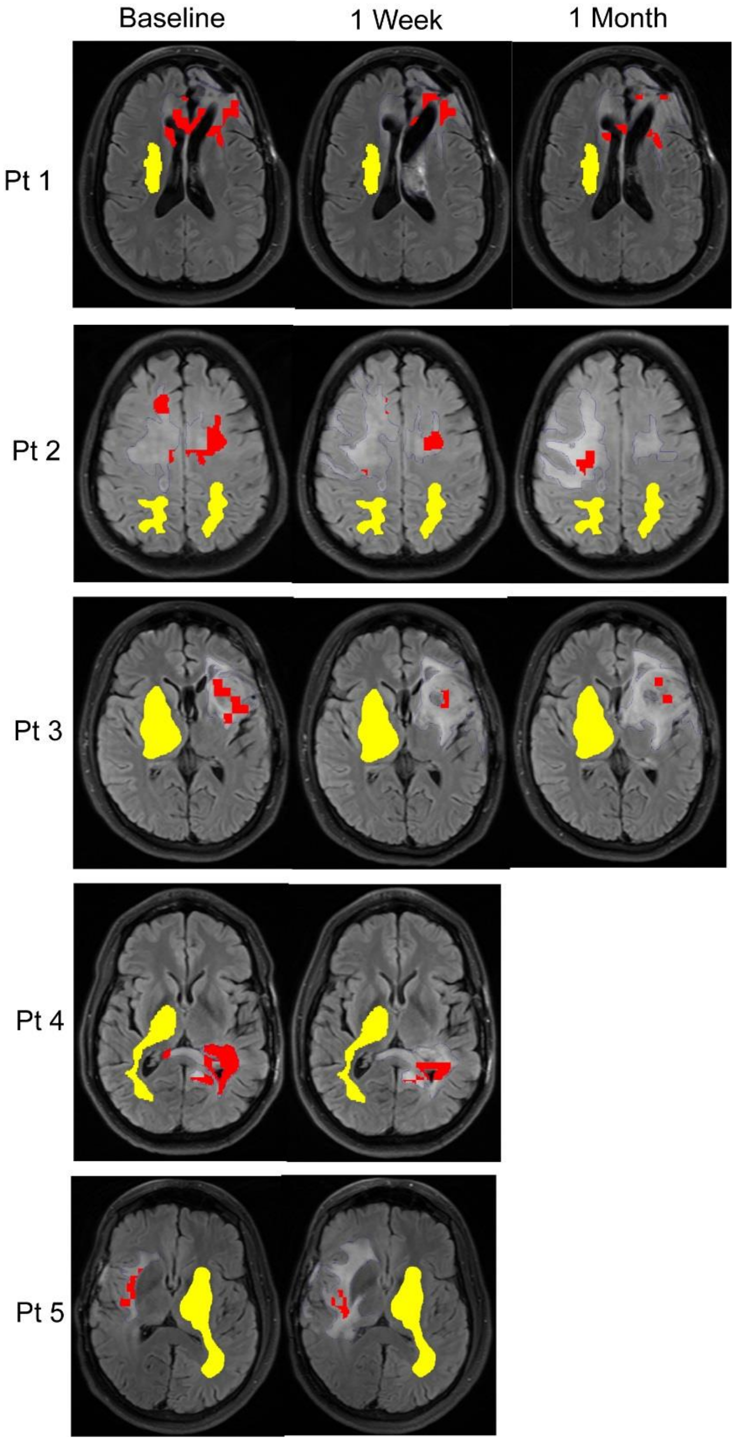

Supplementary Figure 2. Region of interests for image analysis: 1) tumor ROI is outlined by the blue contour around the FLAIR T2 hyperintensity lesion; 2) mask of 2HG voxels with acceptable CRLB goodness of fit is shown in red; 3) healthy ROI in normal appearing white matter on FLAIR is shown by the yellow mask.

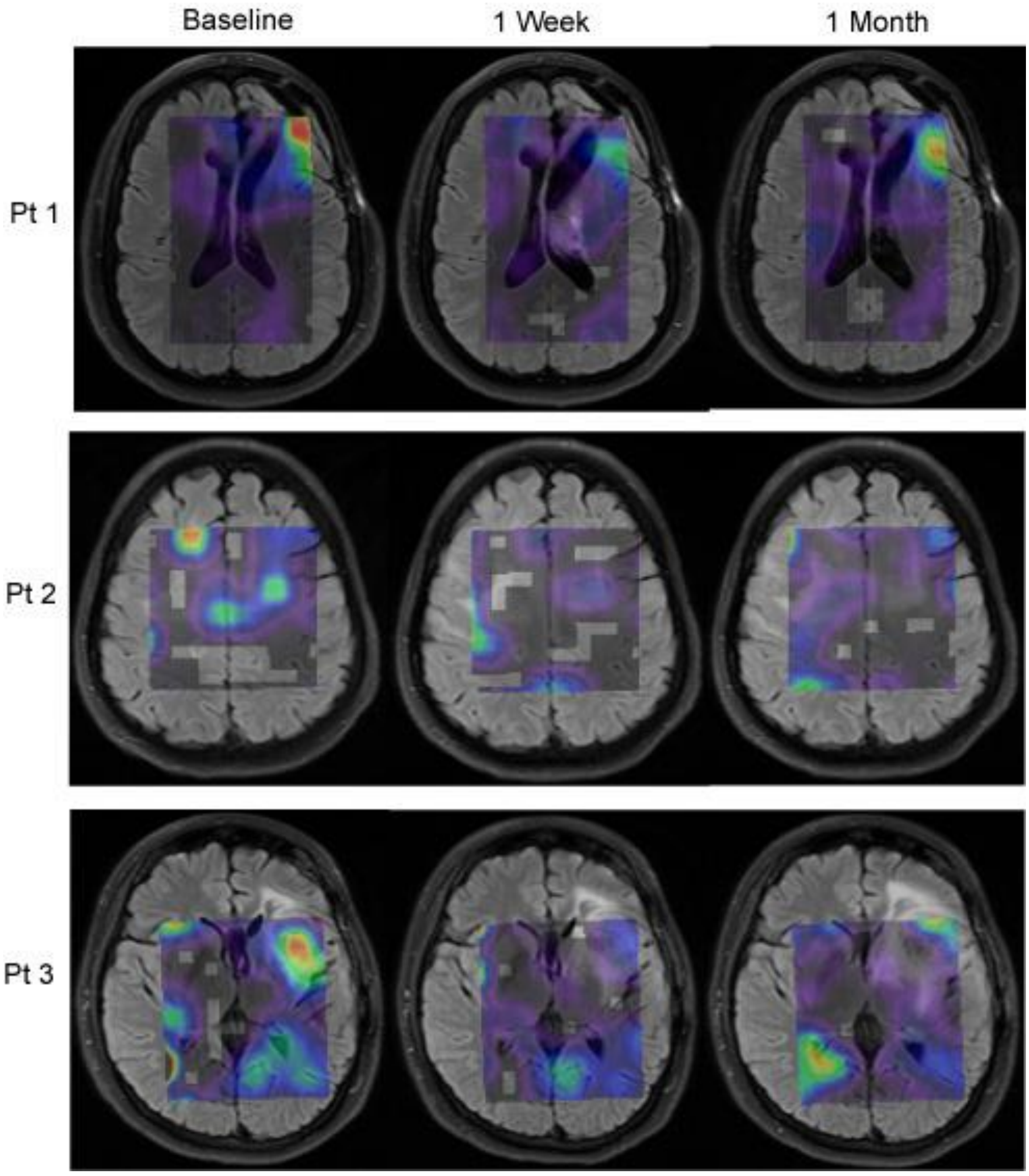

Supplementary Figure 3. 2HG maps for three patients that had a second follow-up scan at 1 month after start of treatment with IDH305 inhibitor of IDH1.

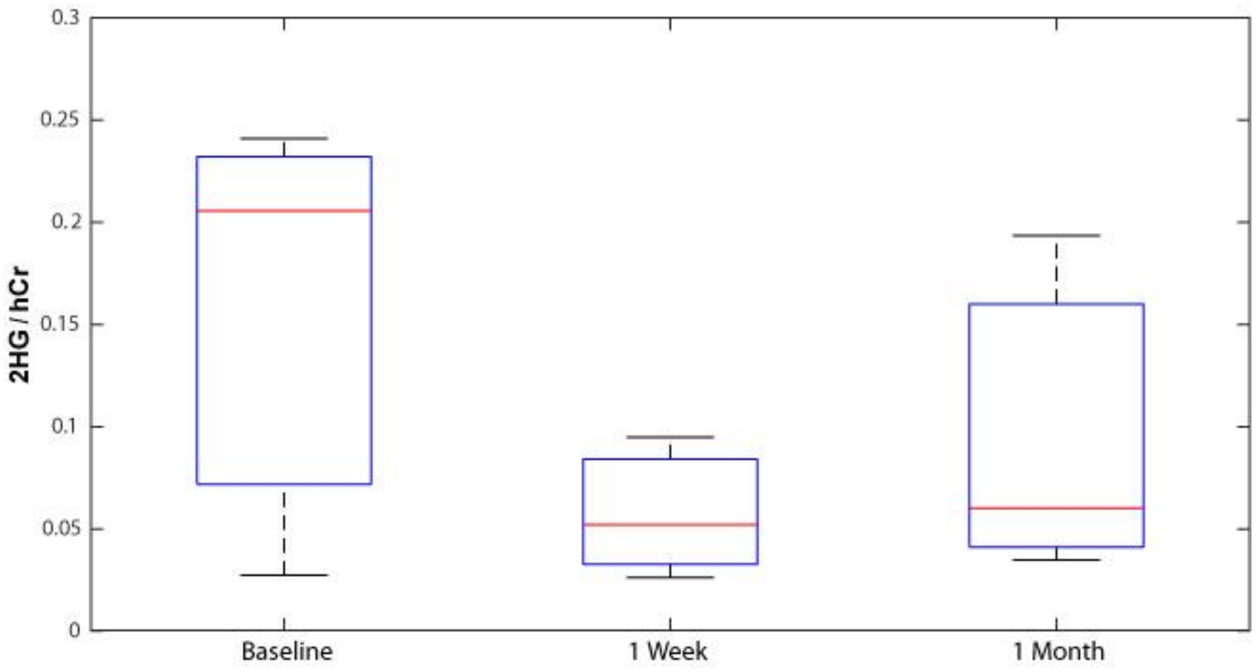

Supplementary Figure 4. Boxplot of 2HG/hCr levels during the first month of IDH305 treatment. 2HG was imaged in three patients for three time points over a month, including baseline (1-5 days pre-treatment), 1 week and 1 month post-treatment scans. Median levels are show by the red line, blue box shows the interquartile range (1st-3rd quartiles), and whiskers indicate the minimum and maximum values.
